# Electronic Measurement-based care (eMBC) for perinatal depression and anxiety: a pilot randomized controlled trial

**DOI:** 10.1186/s12888-025-06876-3

**Published:** 2025-04-29

**Authors:** Neda Askari, Renu Gupta, Neesha Hussain-Shamsy, Lucy C. Barker, Trevor Champagne, Raymond W. Lam, Katie Bishop, Jenna Pirmohamed, Maria Michalowska, Vishva Shah, Hailey Katzman, Ashna Jalan, Anushika Shanmuganathan, Vivienne Wang, Simone N. Vigod

**Affiliations:** 1 Women’s College Hospital, Toronto, ON Canada; 2 Department of Psychiatry, Temerty Faculty of Medicine, University of Toronto, Toronto, ON Canada; 3 Department of Medicine, Temerty Faculty of Medicine, University of Toronto, Toronto, ON Canada; 4 Department of Psychiatry, University of British Columbia, Vancouver, BC Canada

**Keywords:** Perinatal mental health, Perinatal depression, Perinatal anxiety, Measurement-based care, Electronic measurement-based care

## Abstract

**Background:**

As few as 20% of perinatal patients with depression or anxiety are treated to remission. Measurement-based care (MBC) improves patient outcomes but has not been evaluated for perinatal mental illness. We aimed to assess the feasibility of an MBC protocol in perinatal patients experiencing depression and/or anxiety symptoms.

**Methods:**

In this pilot randomized controlled trial (RCT), perinatal people with Edinburgh Postnatal Depression Scale (EPDS) scores *≥* 13 were randomized 1:1 to (1) an electronic MBC (eMBC) intervention embedded in an electronic health record (EHR) that included scales assessing symptoms and functioning at each clinical visit or (2) usual care, for 12 weeks post-randomization. The primary outcome was feasibility (recruitment, acceptability, trial protocol adherence). While not powered to detect clinically significant differences on clinical outcomes, we also measured depressive and anxiety symptoms (Montgomery-Asberg Depression Rating Scale, MADRS; Hamilton Anxiety Scale, HAM-A).

**Results:**

Of 42 participants (*n* = 21/arm), 32 (76.2%) completed follow-up questionnaires. At least one scale was completed in 87.5% of clinical encounters, but only 68.8% of encounters included documented participant-provider discussion of the results. Acceptability was good, with opportunities identified for improvement from participant and provider perspectives. At 12-weeks post-randomization, MADRS and HAM-A scores were non-signficantly lower in the eMBC group (mean differences: -1.10, 95%CI -7.81 to 5.61; -1.28, 95%CI -4.69 to 2.12).

**Conclusions:**

The protocol evaluated in this study was feasible, which supports proceeding to a larger RCT to evaluate efficacy with minor modifications. If effective, an EHR-integrated eMBC intervention for perintal depression and anxiety could be implemented widely.

**Trial registration:**

The trial was registered at www.clinicaltrials.gov (NCT04836585). Registration Date: 08/04/2021.

**Supplementary Information:**

The online version contains supplementary material available at 10.1186/s12888-025-06876-3.

## Introduction

Depression and anxiety affect about 1 in 5 women and gender-diverse birthing persons in pregnancy and postpartum [1]. Left untreated or inadequately managed, perinatal depression and anxiety can have adverse consequences through diminished maternal quality of life, increased risk of substance misuse, self-harm, and suicidal behaviors, and negative impacts on the health and development of children [2–5]. While effective treatment is available, as few as 20% of pregnant or postpartum people achieve remission of their disorders [6] Prompt access to care, intensive monitoring of symptoms, and the ability to more efficiently detect inadequate response to treatment are crucial in the perinatal period given the time-sensitive and time-limited nature of pregnancy and the postpartum.

Measurement-based care (MBC) involves the systematic assessment of clinical indicators (such as symptom severity and functional level) around the time of a clinical encounter to guide treatment decisions. Meta-analyses have shown that incorporating symptom scales into clinical appointments for depression and anxiety can lead to better patient outcomes compared to standard care, particularly for patients who are at risk of inadequate response to treatment, with effect sizes ranging from 0.22 to 0.70 [7–12] Although the low rate of remission for depression and anxiety in perinatal populations is likely to be a result of complex and multi-level barriers, MBC been recommended in clinical practice guidelines for perinatal mental illness to improve treatment response [13]. However, there has yet to be a trial of MBC for perinatal depression and anxiety.

Despite the strong evidence for its efficacy, adoption of MBC in clinical settings has been limited [9]. Barriers to implementation include administrative burden when not embedded within existing documentation pathways [9]. For example, patients may forget to complete paper-and-pen scales prior to and/or to bring them to their appointment, completing symptom scales during the clinical encounter takes up time that could otherwise be spent on treatment, and the time and effort it takes for providers to enter symptom scales into a health record post-encounter could otherwise be spent seeing an additional patient or providing additional treatment. Optimizing information technology capacity to enable patients to seamlessly and digitally complete symptom rating scales prior to their appointment has been suggested as a way to address some barriers [9, 14]. The current study aimed to assess the feasibility of an electronic MBC (eMBC) intervention protocol in a clinical perinatal population experiencing depression or anxiety to determine whether to proceed to a large definitive efficacy trial.

## Methods

### Study design and setting

This was a single-site pilot randomized controlled trial (RCT) with two parallel groups (eMBC and a usual care control group). The study was conducted in a specialized reproductive mental health care clinic at Women’s College Hospital (WCH) in Toronto, Ontario. All perinatal patients referred to the clinic receive a comprehensive psychiatric assessment by one of the program’s perinatal psychiatrists. Together, the patient and psychiatrist formulate a treatment plan, which can include referral to one of the program’s psychotherapy groups, medication and/or other relevant treatments. Patients are followed by the psychiatrist until 12 months postpartum to monitor symptoms and adjust the treatment plan as needed. At the time of this study, the clinic did not use MBC in a standardized manner. All physicians practicing in the clinic agreed to participate in the study.

The electronic REsearch Data CAPture system (REDCap) was used for randomization and management of research data. Data in REDCap were stored on secure servers at WCH. Study participants were identified by a unique study ID number. Qualitative interviews were audio recorded and transcribed. Audio recordings and de-identified transcripts were stored on secure servers at WCH with access limited to designated research staff.

### Interventions

The length of the active treatment phase was 12 weeks post-randomization, aligned with Canadian guidelines on the acute phase of treatment for depressive disorders [15] The experimental condition was electronic MBC (eMBC). MBC comprises 4 core components: (1) a routinely administered symptom, outcome, or process measure (ideally before each clinical encounter); (2) provider review of data; (3) patient review of data; and (4) collaborative re-evaluation of the treatment plan [9] The rationale is that systematic assessment and review of standardized clinical and/or functional outcomes in routine clinical practice will allow improved identification of when a patient is not progressing as desired, and to more timely and effective adjustment of the treatment plan.

For eMBC in the current study, the Edinburgh Postnatal Depression Scale (EPDS) was selected to monitor symptoms of depression and anxiety [16] The EPDS is a 10-item self-report measure that also contains 3 questions focused on anxiety. Although not previously evaluated for MBC purposes to our knowledge, the EPDS is widely used internationally for screening purposes, and familiar to perinatal mental health providers. Aligned with the non-perinatal recommendations for including social functioning in MBC, two brief self-reported scales from the Patient-Reported Outcomes Measurement Information System (PROMIS) assessed functional capacity: PROMIS Neuro-QOL – Ability to Participate in Social Roles and Activities – Short Form, and PROMIS ASCQ-Me Social Functioning – Short Form [17–19] For participants taking antidepressant medication, we also included the 3-item adapted Frequency, Intensity, and Burden Side Effects Rating (FIBSER) Scale to identify concerns about antidepressant side effects [20].

Participants were enrolled in “myHealthRecord,” the secure patient portal of WCH’s electronic health record (EHR) system, Epic. MyHealthRecord was configured to notify participants via email to complete specified scales approximately 24–48 h before each scheduled clinical encounter with their clinic psychiatrist. Participants could also complete scales at e-check-in (which could occur for in-person or video encounters), or at in-person check-in on a tablet in the waiting area. Participants were able to view their responses within their MyHealthRecord account immediately, although they were informed that the scales would be reviewed by their psychiatrist during the visit (i.e., scales were not being reviewed outside of clinic hours). The provider schedule in the EHR was configured so that on the day of the appointment, the psychiatrist could see that the scales had been assigned to their patient, and whether the scales had been completed. The psychiatrist could then review newly- and previously-completed scales in the “Synopsis” tab in the EHR to provide a comprehensive overview of progress over time. The “Synopsis” display allowed for a review item by item and as a total score, in both numerical and graphical formats. The psychiatrist could then review the scales verbally and/or visually with the participant (this could be done via screen-sharing if the encounter was a video-visit).

Both participants and their psychiatrists received a written instruction manual that included technical aspects of the intervention, and for the psychiatrist, a guide to interpreting the eMBC scales (Appendix A). A research staff member provided individualized training sessions via telephone and/or secure video-visit to guide participants in utilizing myHealthRecord to complete symptom scales. Each psychiatrist with a patient randomized to the eMBC group was assisted in turning on a “flag” in the EHR that would automatically send the set of scales to the participants via the patient portal, and in how to configure their daily schedule to be able to automatically view the assignment/completion of the scales. They were also trained on how to view and display the scales in the “Synopsis” tab.

Those in the control condition were followed by their psychiatrist in the clinic. Usual care could involve completion of the EPDS scale or a selection of other symptom scales either by the participant on paper or electronically prior to, or during the clinical encounter (the PROMIS scales were not available). This was not automated, so would be done either by direct provider entry of patient-reported symptoms into flowsheets embedded in the EHR, or by the psychiatrist sending scales in the patient portal to the patient ad hoc.

### Eligibility

Inclusion criteria were: 18 years or older, pregnant or identifying as a mother with an infant between 0 and 12 months of age (through natural birth, adoption, or surrogacy, including cis women, non-binary, and transgender people), and EPDS score *≥* 13. Inclusion criteria were modified during the study to improve implementation of the study procedures. In the initial conception of the project, it was theorized that eMBC would be helpful in particular for patients who needed to decide whether or not to start, continue or change the dose of an antidepressant medication. As such, initial inclusion criteria also required that the psychiatrist apply a DSM-5-TR diagnosis of major depressive disorder (MDD) or generalized anxiety disorder (GAD), either prescribe or recommend an antidepressant, and confirm that at least 3 clinical encounters would be booked over the subsequent 12 weeks. However, after the initial 16 participants, it was determined that psychiatrists were often not able to ascertain any of these criteria until after several appointments had already passed, and treatment had already begun, limiting recruitment feasibility, so these 3 inclusion criteria were removed. Individuals were excluded from the study if they had active suicidality, active alcohol or substance use disorders, current or historical mania or psychosis, or were unable to complete scales in English (no translated versions of the scales were available in the EHR system).

### Recruitment

Potential participants were identified at intake to the clinic where an initial EPDS score is reviewed by the clinic intake worker. If the initial EPDS score was 13 or greater, patients were asked whether they would like to speak to research study personnel about the study. Clinic psychiatrists were also able to identify potential participants from their ongoing patient pool. A dedicated research staff member conducted eligibility assessments and informed consent via telephone, secure video-visit, or in-person meetings.

### Sample size

A sample size ranging from 20 to 40 participants per condition can ensure adequate variability for assessing implementation procedures that would benefit from modification in a future RCT, and for generating feasibility data to support the argument to proceed to a larger RCT [21] As such, we aimed to recruit at least 20 participants/arm, up to a maximum of 80 participants.

### Randomization and blinding

Randomization was conducted within the REDCap platform after baseline measures were completed, with participants assigned in a 1:1 ratio using computer-generated random allocation sequences of randomly fluctuating block sizes ranging from 6 to 8. Each participant was allocated a unique ID, automatically matched to a predetermined and concealed allocation sequence. The research staff member responsible for assigning the unique ID to each participant was not blinded to the treatment allocation, as they had to inform the patient and provider of the allocated treatment and conduct the patient and provider trainings. A different research staff member blinded to the treatment allocation collected outcome measures. Given the nature of the study, participants could not be blinded, although they were not explicitly informed of the study’s hypothesis. Providers were aware of the participant’s allocation, but did not collect outcome data.

### Measures

At baseline, questionnaires administered via REDCap queried data on sociodemographic factors, obstetric, and psychiatric history. A diagnostic telephone interview was conducted using the MINI International Neuropsychiatric Interview to assess MDD, obsessive-compulsive disorder, panic disorder, agoraphobia, social anxiety disorder, GAD, and post-traumatic stress disorder [22].

The primary outcome was the feasibility of the protocol, inclusive of recruitment feasibility, trial protocol adherence and eMBC acceptability. Recruitment measures were the number of participants eligible and recruited. Trial protocol adherence measures were completion of follow-up research questionnaires and adoption of eMBC (number of visits with completed symptom scales and provider review, extracted from the EHR). At 12-weeks post-randomization, a participant acceptability survey was administered to the intervention group comprising 22 closed-ended questions adapted from previous validated surveys on perceived usefulness, ease of use, benefit and satisfaction [23, 24]. Provider perspectives were evaluated through the Provider Perspective Survey, adapted from two studies on provider acceptance of electronic patient records in acute care settings and administered following the collection of participant data [25, 26]. eMBC participants and their providers were also offered to engage in individual semi-structured interviews (30–60 min) to provide further context, in person, via videoconferencing, or telephone depending on participant preference (Appendix B). Patient participants were interviewed after the completing the active treatment phase, psychiatrists when all their patients had completed the active treatment phase.

Secondary outcomes were clinical efficacy outcomes that could be used to measure efficacy in a future large-scale trial. These were collected at 12 weeks post-randomization by a trained research staff member by telephone, secure video-visit and/or in-person, as per patient preference. The primary efficacy measure was the Montgomery-Asberg Depression Rating Scale (MADRS), a standard clinician-administered tool for assessing depressive symptoms [27] Widely recognized for its reliability and validity in clinical settings, the MADRS comprises 10 items rated on a 7-point scale, yielding a score range of 0–60. It demonstrates good responsiveness to the effects of antidepressant treatment. A MADRS score of < 10 is considered remission. Additionally, the Hamilton Anxiety Scale (HAM-A) was utilized to assess the severity of anxiety symptoms. This clinician-rated scale, consisting of 14 items, is known for its reliability and validity in populations affected by depression and anxiety [28] Ratings are based on a 5-point scale, resulting in a score range of 0–56. A HAM-A score of < 8 is considered anxiety remission.

### Data analysis

We calculated proportions and means for baseline characteristics, and to assess recruitment, trial protocol adherence outcomes and acceptability questionnaires. In an intention-to-treat analysis, MADRS and HAM-A means were compared between groups at 12-weeks post-randomization using analyses of covariance (ANCOVA) with baseline score as the covariate, generating adjusted mean differences (aMD) and 95% confidence intervals (CI). We also compared the proportion achieving remission at 12 weeks post-randomization between groups on these two measures using modified Poisson regression to generate relative risks (RR) and 95%CI. Given the small expected numbers we did not plan additional analyses to account for missing data. Qualitative analysis involved analyzing interview transcripts using thematic analysis [29]. This included: reading and re-reading transcripts to gain a comprehensive understanding; closely examining transcripts and assigning initial codes; grouping codes into potential themes; reviewing themes and creating a thematic ‘map’ of the analysis; and refining themes and organizing them into main themes/sub-themes. A hybrid approach, where a small codebook was a priori created based on the research aims, and new codes were created as needed while transcripts were being examined (i.e. both deductive and inductive approaches) was used [30]. This helped ensure codes and subsequent themes were relevant to the overall purpose of this study, while maintaining freedom to highlight other pertinent information relevant to participants during the analysis. Quantitative analysis was conducted using SPSS v. 29.0.1.0 and qualitative analysis using NVIVO software.

### Ethical considerations

The trial was registered at www.clinicaltrials.gov (NCT04836585), registration date 08/04/2021. Approval for the study was obtained from the Research Ethics Board at Women’s College Hospital in Toronto, Ontario (Approval # 2020-0090-B). Participants received nominal tokens of appreciation in the form of gift cards for completing follow-up assessments and qualitative interviews.

## Results

**Fig. 1:**
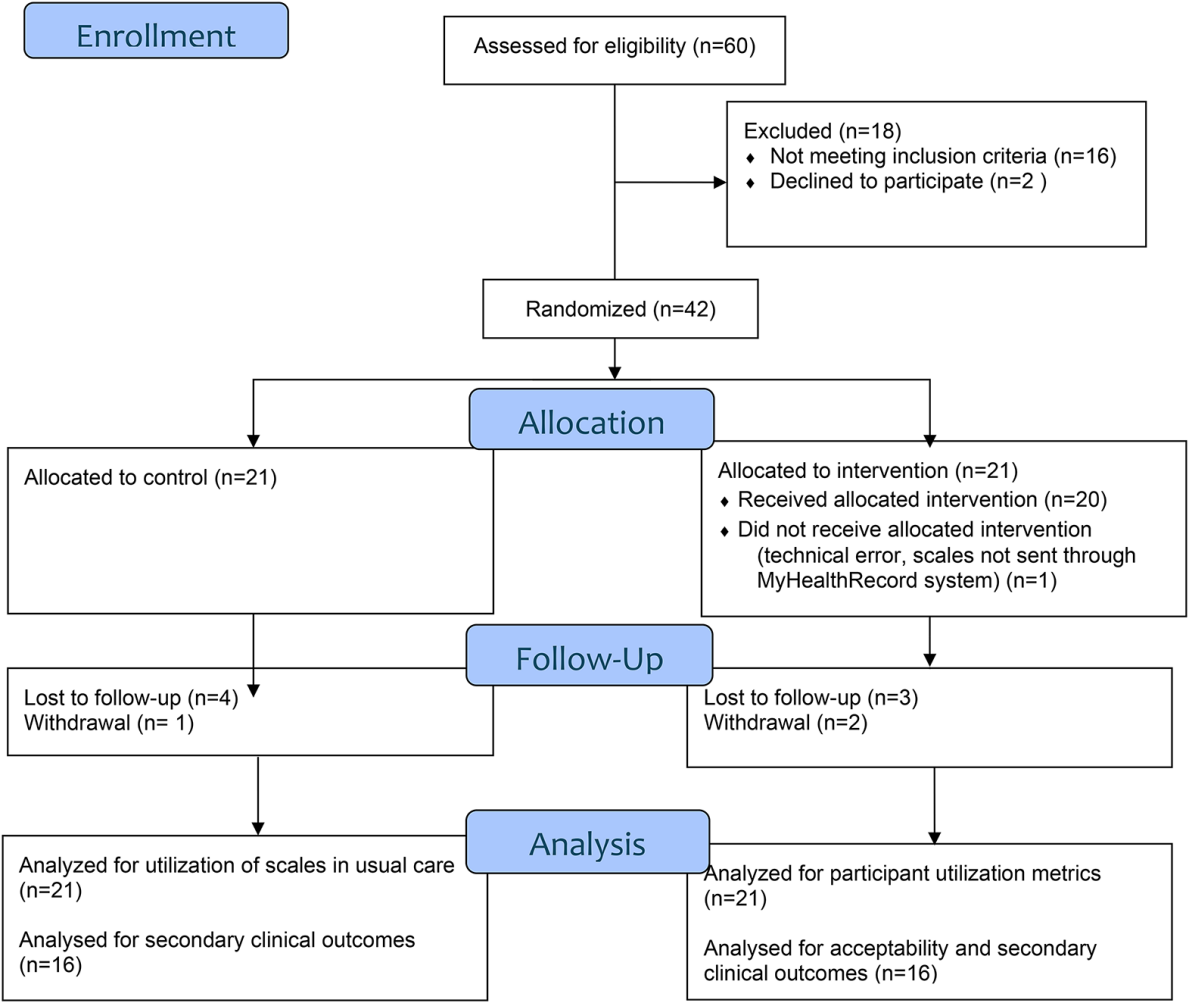
CONSORT diagram for eMBC for Perinatal Depression and Anxiety study

Participants were recruited over 18 months (January 2022 to July 2023) with final follow-up in October 2023. Of 60 participants assessed for eligibility, 16 did not meet the inclusion criteria and 2 declined participation, resulting in 42 participants randomized to either eMBC (*n* = 21) or the control group (*n* = 21)(Fig. 1). Participants ranged in age from 25 to 44 years and about half were pregnant at the time of enrollment. Most participants were highly educated, and had a partner, with family income greater than $80,000 CAD per year. Most participants had a history of depressive disorder. About one-fifth met criteria for a MDD diagnosis and about half for a GAD diagnosis at enrollment (Table 1).

**Table 1:** Baseline characteristics for eMBC participants and controls, presented as N (%) unless otherwise specified

Variable	eMBC (*N* = 21)	Control (*N* = 21)
Demographics		
Mean (SD) age in years	34.0 (3.5)	34.0 (4.5)
Pregnant at enrolment	11 (52.4)	10 (47.6)
Marital Status (Married or Cohabitating)	19 (90.5)	19 (90.5)
Median (IQR) Number of children in the home	1 (1–2)	1 (0–2)
Sexual orientation (Heterosexual)	17 (81.0)	15 (71.4)
Highest level of education (university/college completed)	19 (90.5)	19 (90.5)
Annual household income (>$80,000 CAD)	18 (85.7)	17 (81.0)
Immigrant to Canada	7 (33.3)	5 (23.8)
Mean (SD) years since immigration	12.9 (9.2)	19.6 (10.1)
Language spoken other than English/French	7 (33.3)	10 (47.6)
Psychiatric Profile		
Major Depressive Disorder (any history)	20 (95.2)	17 (81.0)
Prior psychiatric hospitalization	1 (4.7)	2 (9.5)
Prior suicide attempt	3 (14.3)	2 (9.5)
Currently taking medication for mental health concerns	11 (52.4)	17 (81.0)
Baseline Mean (SD) MADRS	22.3 (6.7)	20.4 (8.0)
Baseline Mean (SD) HAM-A	10.3 (5.9)	10.6 (5.8)

### Feasibility outcomes

In the eMBC group, 20 received the allocated intervention; one did not receive the allocated intervention due to technical errors. Follow-up questionnaires were completed by 16 out of 21 (76%) of participants at 12-weeks post-randomization in both groups. There were 12 psychiatrists who had participants randomized to eMBC, but 3 were investigators on the protocol (RG, SV, LB), leaving 9 providers eligible to fill out the provider survey, 8 of whom did so (Table S1 for baseline characteristics). Eight participants and four of the psychiatrists also participated in optional qualitative interviews.

In the eMBC group, there were 48 total clinic visits within 12 weeks (median of 2.5, interquartile range (IQR) 2 to 3 per person). There was at least one scale filled out in 87.5% of the visits, and each of the mandatory scales (EPDS and the 2 PROMIS scales) were filled out in about 80% of visits (Table 2). However, psychiatrists documented on having a discussion with the patient about the scales in only 68.8% of the visits. A chart review of the 21 patients in the usual care group revealed that none were administered any symptom or functional scales during the 12-week post-randomization follow-up window.

**Table 2:** Participant utilization metrics (intervention group only, *n* = 21), for 48 total clinic visits within 12 weeks post-randomization. Values are provided as N (%), unless otherwise specified

	*N* (%)
**By clinical encounter (** *n* ** = 48)**	
Mandatory scales filled out:	
Any scale filled out	42 (87.5)
One scale filled out	0 (0)
Two scales filled out	8 (16.7)
Three scales filled out	34 (70.8)
**Type of scale filled out**	
EPDS	40 (83.3)
PROMIS Neuro-QOL	39 (81.3)
PROMIS Social functioning	38 (79.2)
FIBSER (optional, only if taking antidepressant)	18 out of 31 (58.0)
**Chart Documentation**	
Patient-provider discussion about scales	33 (68.8)
Any change in treatment	10 (20.8)
Start medication	3 (6.4)
Change medication dose	16 (33.3)
Switch medication	2 (4.2)
**By participant (** ***n*** ** = 21)**	
Completed at least 1 scale	19 (90.5)
Completed at least 1 scale in 50%+ of visits	18 (85.7)
Completed all 3 mandatory scales in 50% of visits	15 (71.4)
Completed all 3 mandatory scales in all visits	11 (52.4)

*Note*: Encounters not included in the denominator due to technical difficulties: For eMBC 6 encounter #1, participant reported scales completed, but they were not in the EHR; For eMBC 42 all encounters and eMBC 30 encounters #2 and #3, participant indicated that they did not receive the scales

### Participant and psychiatrist perspectives

Participant views of the intervention were very positive (Table 3). More than 80% liked the idea of using eMBC, found it easy to become skillful to use it, and thought that using eMBC made them more actively involved in their mental health. All (100%) of respondents thought that using eMBC can or should be recommended to those who are in a similar condition to them. All psychiatrists agreed that eMBC was easy to learn, a good idea for the monitoring patients and that they intended to use it when it became more widely available in the clinic (Table 3). Yet, some expressed concerns using eMBC would require significant changes to their practice, and that it would not be easy to perform the tasks necessary to monitor their patients using it. Three major themes emerged from participant and psychiatrist qualitative interviews, all related to MBC in the context of the patient journey: (1) eMBC as a part of a patient-centred journey, (2) acceptability of MBC as a tool within the context of clinical care, and (3) implementation feasibility (Table 4). No adverse events were reported over the course of the study.

**Table 3:** Participant (intervention group only, *n* = 16) and provider (*n* = 8) acceptability in N (%), with shaded grey representing positive views of eMBC

	AGREE			DISAGREE		
	**STRONG**	**MOD**	**MILD**	**MILD**	**MOD**	**STRONG**
**Participant Questionnaire (** ***n*** ** = 16)**						
Using eMBC has helped improve my mental health problem.	2 (12.5)	2 (12.5)	7 (43.8)	2 (12.5)	3 (18.8)	0
Using eMBC has improved my ability to manage my mental health problem.	2 (12.5)	3 (18.8)	6 (37.5)	2 (12.5)	3 (18.8)	0
It was easy for me to become skillful at using eMBC through MyHealthRecord.	5 (31.8)	3 (18.8)	5 (31.8)	1 (6.3)	2 (12.5)	0
I like the idea of using eMBC.	4 (25.0)	6 (37.5)	3 (18.8)	1 (6.3)	2 (12.5)	0
I predict that I will use eMBC in the future.	3 (18.8)	3 (18.8)	6 (37.5)	2 (12.5)	2 (12.5)	0
Completing the eMBC scales has interfered with my everyday routine.	1 (6.3)	0	3 (18.8)	1 (6.3)	4 (25.0)	7 (33.3)
eMBC has been explained to me sufficiently.	8 (38.1)	4 (25.0)	0	2 (12.5)	2 (12.5)	0
eMBC can be trusted to work appropriately.	6 (37.5)	6 (37.5)	1 (6.3)	1 (6.3)	1 (6.3)	0
eMBC has invaded my privacy.	1 (6.3)	0	0	0	3 (18.8)	11 (52.4)
eMBC made me feel uncomfortable (e.g. physically, emotionally)	1 (6.3)	0	0	1 (6.3)	1 (6.3)	13 (61.9)
eMBC makes me worried about the expertise of those who monitor my mental health status via MyHealthRecord.	1 (6.3)	0	1 (6.3)	1 (6.3)	3 (18.8)	10 (62.5)
eMBC allowed me to be less concerned about my mental health and/or care.	1 (6.3)	3 (18.8)	5 (31.8)	2 (12.5)	1 (6.3)	4 (25.0)
eMBC has made me more actively involved in my mental health.	3 (18.8)	6 (37.5)	4 (25.0)	2 (12.5)	0	1 (6.3)
eMBC makes me worried about the confidentiality of my private info being exchanged through MyHealthRecord.	1 (6.3)	0	0	1 (6.3)	3 (18.8)	11 (52.4)
eMBC allows the people who provide care to me, to better monitor me and my mental health problem.	9 (56.3)	4 (25.0)	1 (6.3)	0	2 (12.5)	0
eMBC can/should be recommended to people in a similar condition to mine.	5 (31.8)	4 (25.0)	7 (43.8)	0	0	0
eMBC can certainly be a good addition to my regular care.	5 (31.8)	5 (31.8)	4 (25.0)	1 (6.3)	1 (6.3)	0
eMBC made it easier to get in touch with my psychiatrist.	4 (25.0)	6 (37.5)	4 (25.0)	0	1 (6.3)	1 (6.3)
eMBC has allowed me to be less concerned about my mental health problem.	2 (12.5)	2 (12.5)	7 (43.8)	1 (6.3)	1 (6.3)	3 (18.8)
I am satisfied with eMBC.	6 (37.5)	2 (12.5)	6 (37.5)	2 (12.5)	0	0
People who influence my behaviour think that I should use the eMBC system.	1 (6.3)	3 (18.8)	4 (25.0)	5 (31.8)	2 (12.5)	1 (6.3)
People who are important to me think I should use the eMBC system.	0	5 (31.8)	4 (25.0)	5 (31.8)	1 (6.3)	1 (6.3)
**Provider Questionnaire (** ***n*** ** = 8)**						
I feel comfortable with information and communication technologies.	1 (12.5)	4 (50.0)	3 (37.5)	0	0	0
The use of eMBC could help me monitor my patients more effectively.	4 (50.0)	2 (25.0)	1 (12.5)	0	1 (12.5)	0
eMBC is easy for me to learn.	3 (37.5)	1 (12.5)	4 (50.0)	0	0	0
I think it is a good idea to use eMBC to monitor my patients.	3 (37.5)	4 (50.0)	1 (12.5)	0	0	0
I have the intention to use eMBC when it becomes available at WCH.	6 (75.0)	1 (12.5)	1 (12.5)	0	0	0
The use of eMBC may require significant changes in my existing clinical practice.	1 (12.5)	2 (25.0)	0	3 (37.5)	1 (12.5)	1 (12.5)
I think it would be easy to perform the tasks necessary for the monitoring of my patients using eMBC.	0	3 (37.5)	2 (25.0)	2 (25.0)	1 (12.5)	0
Most of my patients will welcome the fact that I use eMBC.	1 (12.5)	3 (37.5)	2 (25.0)	2 (25.0)	0	0
I think that WCH has the necessary infrastructure to support the use of eMBC.	1 (12.5)	4 (50.0)	0	0	3 (37.5)	0
eMBC could help me get the most out of my time to monitor my patients.	2 (25.0)	2 (25.0)	3 (37.5)	1 (12.5)	0	0
I believe that the monitoring carried out by eMBC would be clear and easy to understand.	1 (12.5)	5 (62.5)	2 (25.0)	0	0	0
The use of eMBC is compatible with my work habits.	1 (12.5)	3 (37.5)	3 (37.5)	1 (12.5)	0	0
Most of my colleagues will welcome the fact that I use eMBC.	2 (25.0)	3 (37.5)	3 37.5)	0	0	0
eMBC can improve my performance in delivering care.	3 (37.5)	1 (12.5)	2 (25.0)	1 (12.5)	1 (12.5)	0
eMBC may promote good clinical practice.	3 (37.5)	2 (25.0)	3 (37.5)	0	0	0
I think that eMBC is a flexible technology to interact with.	2 (25.0)	1 (12.5)	3 (37.5)	2 (25.0)	0	0
I find it interesting to use eMBC for the monitoring of my patients.	3 (37.5)	3 (37.5)	1 (12.5)	1 (12.5)	0	0
I have already used telemonitoring devices to monitor my patients.	2 (25.0)	3 (37.5)	3 (37.5)	0	0	0
The use of eMBC is beneficial for the care of my patients.	3 (37.5)	3 (37.5)	2 (25.0)	0	0	0
I would use eMBC if I receive appropriate training.	4 (50.0)	2 (25.0)	1 (12.5)	1 (12.5)	0	0
The use of eMBC may interfere with delivering care.	0	1 (12.5)	2 (25.0)	2 (25.0)	3 (37.5)	0
In my opinion, the use of eMBC will have a positive impact.	2 (25.0)	4 (50.0)	2 (25.0)	0	0	0
I often use computing tools in my work.	5 (62.5)	1 (12.5)	2 (25.0)	0	0	0
Using eMBC does not fit the way I view mental health care.	0	0	1 (12.5)	5 (62.5)	0	2 (25.0)
Using eMBC runs counter to my values about how to care for my patients.	0	0	1 (12.5)	3 (37.5)	2 (25.0)	2 (25.0)

**Table 4:** Qualitative interview results

**Theme 1: eMBC as a part of a patient-centred journey**
***Theme 1*** followed the journey of patients as completing scales helped them acknowledge their own mental health status. For example, they used the task of completing the eMBC scales as an opportunity to reflect: “*The scales actually were quite helpful*,* because sometimes you don’t realize that you’re feeling the way you are until you kind of sit down and have a moment to reflect*” (Participant 035). This theme included the idea that completed eMBC scales could be “a point of reference to start the conversation” (Participant 056) between patients and their healthcare providers. However, eMBC could also cause frustration if the scales were not considered or discussed during the patient-provider consultation. As one patient said: “*The results weren’t really talked about… nothing really came out of it… I was disappointed. And then*,* when I did it the next time*,* it was the same thing and I was like*,* this is really tedious and not useful to me* (Participant 029).” When used, MBC could support monitoring of clinical progress: “*I think that it has been beneficial to have the objective measures…It has been helpful to kind of reflect back to them. Hey*,* look*,* your score was this last time*,* and now it has come to something else.*” (Provider 06). Sometimes, the questionnaires may have provided insight beyond the clinical encounter: “*Sometimes… I thought they were doing better*,* my clinical impression*,* and then their score may have indicated something that was lower*,* so I guess there was room to discuss that*” (Provider 02).
**Theme 2: Acceptability of MBC as a tool within the context of clinical care**
***For Theme 2***, acceptability of MBC as a tool within the context of clinical care, providers felt that eMBC had the potential to be a helpful tool for monitoring symptoms and functioning. However, one provider also indicated: *“…to wholistically evaluate how a person is doing*,* there is nuance and important pieces that aren’t captured in the rating scales that come about better within the rapport and safety of the therapeutic relationship. So*,* I don’t think they are helpful to use in isolation either*,* but I do think they can be one component of an assessment that helps to guide our treatment*” (Provider 03). Some providers questioned whether the specific scales were appropriate for all patients; eMBC might not be appropriate based on that patient’s context (i.e. social and medical history, personality, past experiences with the medical system). One provider noted: “*Some people that have trauma backgrounds have been more sensitive*,* but that wasn’t everyone. But certainly there are some people I think that were maybe more sensitive and interpersonally didn’t want to feel… reduced to a number* (Provider 02).” One participant also made a comment in this regard: “*For someone who is ADHD and on the spectrum*,* the ASD*,* it’s harder to answer*,* like*,* yes or no sort of questions or*,* like*,* put it on a scale… specifically for people that are neurodiverse*,* these type of questionnaires are very difficult to answer… I see how it could benefit other people*,* but it didn’t specifically benefit me.*” (Participant 029).
**Theme 3: Feasibility of the implementation of eMBC**
The final theme centred on issues related to the implementation of eMBC, particularly from a technology and training perspective. Participants appreciated online access but indicated that it could be frustrating if the system did not work as expected (e.g. if the scales did not get delivered prior to the clinic appointment and had to be completed during consultation time). Providers appreciated the automated nature of eMBC “… *saves me from having to make sure that I’m tracking them*,* sending the right questionnaires to the patient directly because that is already done for us*.” (Provider 03). Patient participants felt the training provided was adequate, but providers expressed a need for more direct, in-person training: “*I think… sitting down with somebody and showing me*,* it’s easier for me to figure out something than doing it virtually. And then if you needed to ask*,* there isn’t really somebody*,* and then the patient is already there on the video. I think that was the biggest barrier*” (Provider 04).

### Clinical outcomes

Mean MADRS decreased from 22.2 (standard deviation, SD, 6.89) to 12.3 (SD 11.7) in the eMBC group and from 21.8 (SD 9.74) to 13.0 (SD 9.74) in the usual care group at 12 weeks post-randomization, an aMD of -1.10 points (95% CI -7.81 to 5.61). About 56.4% (*n* = 9 out of 16) in the eMBC group had MADRS score < 10 (depression remission) compared to 43.8% (*n* = 7 out of 16) of in the usual care group at endpoint (RR 1.29, 95%CI 0.64–2.60). Mean HAM-A score decreased from 10.9 (6.38) to 6.63 (6.69) in the eMBC group and 11.1 (5.61) to 8.06 (5.03) in the usual care group at 12 weeks post-randomization, corresponding to an aMD of -1.28 points (95%CI -4.69 to 2.12). About 75% (*n* = 12) of the eMBC group had HAM-A score < 8 (anxiety remission) compared to 56.3% (*n* = 9) of the usual care group at this time (RR 1.30, 95%CI 0.80–2.24).

## Discussion

In this pilot RCT of a novel protocol to evaluate eMBC for perinatal depression and anxiety, target recruitment was met, the intervention was acceptable to participants and providers, and research follow-up neared 80%. Preliminary efficacy analyses trended in a positive direction for depression and anxiety symptoms. Opportunities for improvement for a future RCT include further optimizing technical infrastructure, exploring how to improve provider buy-in, and, since most participants only had 2 or 3 clinical encounters over the 12-week post-randomization period, potentially extending the outcome period to allow for a more comprehensive estimation of intervention effects.

The study results are very consistent with evaluations in non-perinatal populations, particularly with regard to the high acceptability of technology-based MBC among patients, where eMBC data collection’s more flexible, usable, and convenient self-assessment platform appears to best facilitates monitoring of symptoms [31]. An important strength of the current study is that eMBC was embedded in the clinic’s EHR, allowing for a streamlined approach for both patients and psychiatrists that may have further enhanced acceptability. Our results are also consistent with literature where patient acceptability is high among participants younger than 40 years old, due to their overall comfort with technology [32]. In the current pilot, where our sample of perinatal patients had a mean age of 34, most participants readily adopted the eMBC intervention and found it acceptable for use. This is the first time to our knowledge that the EPDS has been tested in an eMBC trial, supporting acceptability for its use in measurement-based care. Also similar to non-perinatal literature, the psychiatrists in this study held mostly favourable views, but also had some concerns about eMBC [33].

Learnings from this study can be used to inform both a future RCT to evaluate efficacy and other work on implementation of eMBC in the perinatal mental health context. Some participants and providers felt that the symptom scales delivered through the eMBC intervention didn’t fully “represent” the patient experience. Adding routine scales that capture additional types of symptomatology (e.g., post-traumatic stress, obsessive-compulsive) could be considered, but patient burden in completing scales is also a key factor [34]. Another modification to consider is whether to include an arm in a future study that allows a more flexible selection of symptom scales - collaboratively decided on by patient and provider. This might improve uptake and adherence in real-world implementation, so evaluating whether it is as effective as implementing pre-specified standard scales could be of interest. Finally, participant and provider training could incorporate more about the purpose of eMBC and the reasons for including specific scales. Specifically, ensuring training materials are more clear that the scales are meant to function as a starting point and guide for conversations with their psychiatrist, not to capture all symptoms.

One key finding of this pilot study was that the psychiatrists only documented discussion about the scales in about two-thirds of clinical encounters (whereas scales were completed by the patient over 80% of the time). Patients understandably expressed disappointment when results were not discussed. There are several potential reasons why providers may not have reviewed the scales with their patients. Some psychiatrists expressed a desire for more hands-on training on how to access and display the scales in the EHR. In our protocol, psychiatrists received a training manual and could optionally receive additional hands-on training virtually or in-person. Provider training for a future trial could also include simulations around how to use the scales to guide conversation and overtly acknowledge patient effort in completing them [35]. However, in real-world implementation, intensive training might not be realistic, and most psychiatrists did indicate that they found the eMBC easy to use. It may be that other barriers are more relevant. For example, although all psychiatrists felt eMBC was a good idea, a few indicated that eMBC would result significant changes to their practice. Some expressed concerns that the tools might cause increased distress for certain patients, might not be applicable to certain patients, or might not be essential parts of care. In other studies, barriers to eMBC uptake include provider beliefs that measures are no better than clinical judgments and worries that MBC data could be used to judge their therapeutic skillfulness [9]. Identifying MBC champions to reinforce adoption is a strategy suggested to ameliorate provider concerns [9]. The positive participant views of eMBC in our study (not known to the providers at the time that they completed their own surveys and interviews) might also be helpful in addressing provider concerns, improving adherence to the full eMBC model in a future larger-scale trial.

Limitations of this work are acknowledged. In the 12-week active treatment period, there were, on average, only 2 or 3 visits per patient. It might be wise in a future trial to extend measurements to a 24-week post-randomization period for efficacy measurement to allow for a greater opportunity for treatment decisions to be operationalised and take effect, and for clinical effects to be maintained. Additional limitations include the relatively high-income nature of the participants, which, although common in clinical studies, does raise questions about implementing the eMBC protocol in lower-resource populations (particularly those who do not have as ready access to the internet to complete scales prior to their clinical encounter). A future trial, possibly multi-site, could make efforts to include a more representative sample from a socio-economic status perspective.

## Conclusions

Despite the large body of evidence supporting MBC for improving depressive and anxiety outcomes, this appears to be the first published work on the use of eMBC to support the delivery of perinatal mental health services. This study demonstrates that, with minor adjustments, it will be feasible to implement a protocol to assess the efficacy of eMBC embedded in a real-world electronic health record for perinatal depression and anxiety. If efficacious, eMBC could be used as part of routine clinical care to improve outcomes for perinatal individuals with depression and anxiety.

## Electronic supplementary material

Below is the link to the electronic supplementary material.

Supplementary Material 1

Supplementary Material 2

Supplementary Material 3

Supplementary Material 4

## Data Availability

The datasets analysed during the current study are available from the corresponding author on reasonable request, and may require the establishing of data sharing agreements according to the author’s institutional and research ethics board requirements.
