# Supplementary material for: Electronic Measurement-based care (eMBC) for perinatal depression and anxiety: a pilot randomized controlled trial
Source: BMC Psychiatry. 2025 Apr 29;25:437. doi: 10.1186/s12888-025-06876-3 (PMC12042506; doi:10.1186/s12888-025-06876-3)
Supplement: Supplementary file 1 — Supplementary Material 1 [file 12888_2025_6876_MOESM1_ESM.docx]

Appendix B: Provider and Patient Qualitative Interview Guides

eMBC Qualitative Interview Guides for Providers v2; 04-Apr-2023; Qualitative Interview Guides for Patients v2; 04-Apr-2023

Contents

[eMBC Qualitative Interview Guide for Providers 2](#_Toc185429565)

[Set Up 2](#_Toc185429566)

[Interview Discussion 3](#_Toc185429567)

[Wrap up 5](#_Toc185429568)

[eMBC Qualitative Interview Guide for Patients 6](#_Toc185429569)

[Set Up 6](#_Toc185429570)

[Interview Discussion 7](#_Toc185429571)

[Wrap Up 10](#_Toc185429572)

# eMBC Qualitative Interview Guide for Providers

**Implementation of technology-enabled (Electronic) Measurement Based Care (eMBC) for Perinatal Depression and Anxiety: Qualitative Study**

Interview Guide – Physician Participants

The interview guide was adapted from a template created by (Cuperfain et al., 2021).

**Venue**: Interviews will be conducted at Women’s College Hospital, by telephone, video or in-person, whichever is most convenient and allows for sufficient privacy The exact time and location will depend on room availability and will be communicated to participants in advance.

**Duration**: Interviews will last approximately 30-60 minutes or as long as it takes for participants to complete their thoughts. If the participant is tried, let them take a break.

**Number of Facilitators**: Each interview will be conducted by a member of the research team.

## Set Up

1. Introduce yourself and the purpose of the interview, e.g., “I am a physician/investigator/research assistant working on a research study about electronic Measurement Based Care (eMBC), a new model of care that involves the regular completion of symptom scales to help improve the treatment of depression and anxiety. The main purpose of this interview is to understand your perspective and experience with electronic measurement-based care or eMBC to ensure it is best suited to help people with depression and anxiety in pregnancy and postpartum.
2. Review the key content in the consent form (e.g., confidentiality and anonymity, the participant’s right to withdraw and to delete data) and offer to answer any questions the participant may have. Ensure that the consent forms have been signed for each participant.
3. Explain the need for audio-recording and obtain approval from all participants. [Remember to bring your recorder and to check it for proper functioning, including sufficient battery life, memory space for recording].

When the audio-recorder is turned on, state “For the purpose of the recording, I would like to state that today is *(date)* and this is my *(time)* interview with *(participant ID).*”

## Interview Discussion

**1. Background**

1. Please tell us a little bit about your background, years in practice and about your prior experience with MBC prior to this pilot trial?

**2. Open exploration**

1. Start with a brief prompt
   1. Thank you for taking the time to participate in this interview. We are most interested in your personal experience, perspective and feelings on eMBC, and in particular the eMBC implementation in perinatal care. You can start with whatever you want to talk about first.
2. The main purpose of the open exploration part is to allow participants to freely express themselves. The participant should decide what they want to talk about based on what is important to them.
3. Use more probes to invite participants to elaborate on certain topics.
4. Try and jot down notes during the interview to help keep track of what has been said
5. Once you believe the open exploration is complete, try to summarize key points of the conversation and thank them for sharing.
6. Introduce the participants to the structured exploration part of the interview: “Next, I have a few specific questions I am going to ask about your experience and perspective on eMBC”

**3. Structured inquiry:** Ask participants about specific features of eMBC. For each feature, remember to encourage the “think aloud” method and ask:

- *How do you feel when you see this?*
- *What was your initial reaction to this?*
- *What do you think this feature/content is trying to accomplish?*

*Note: Before asking any questions, note if any of them have been answered during the Open Exploration. Only ask questions that have not been previously addressed.*

- 1. In general, what are your experiences and comfort level with technology?
     1. Have you used virtual or web-based treatments before? If so, which type of treatment and how was your experience?
  2. What were your experiences with MBC like prior to this study, either electronic or non-electronic? [If not already answered in background]
  3. What were your initial reactions to eMBC? What were your thoughts about using eMBC in your own clinical practice?
  4. What are your views on the goals of eMBC?
     1. What are your thoughts on whether eMBC will impact patient experiences and outcomes?
  5. How was your experience learning how to use eMBC (i.e., viewing results of symptoms scales on EPIC)?
     1. What are your thoughts about the training you received?
     2. Do you have any suggestions for how we can improve training?
  6. How did you introduce eMBC to your patients? What were their reactions?
     1. If patients declined, why?
  7. Overall, how was your experience using eMBC?
     1. What were the greatest benefits in utilizing eMBC?
        - Do you think eMBC in any way facilitated your patients’ treatment or recovery?
     2. What were the greatest challenges in utilizing eMBC?
        - How did you navigate these challenges?
        - What would have helped address any challenges you may have faced in utilizing eMBC?
        - Were there any particular characteristics of this patient population that may have been a factor?
        - Any technical issues?
     3. What are your views on possible risks of eMBC?

Here are the questionnaires from the study.

What is your opinion on the scales? Which ones did you find useful? Which ones were not useful?

Did you find that some symptoms better captured than others? If so, which?

What is your opinion on the synopsis visual tracking of patient scores?

Did you go through the scales with your patients?

- - - - How did you go about this? What was your experience like?

How long did it take? If you did not go through the scales, why?

- 1. What was the general response towards eMBC among your patients?
     1. Was eMBC burdensome for your patients?
     2. Do you feel that eMBC hindered the clinical encounter at any point?
  2. After using eMBC, were there any changes in your perspectives?
     1. Does your opinion differ by diagnosis?
  3. Do you think you will use eMBC in your practice going forward? Why or why not?
  4. Do you have any other suggestions about how eMBC could best be integrated with the care you deliver?
     1. What, if anything, would you like to see done differently in eMBC (e.g., content, delivery, etc.)?
     2. What would be needed for this to be a sustainable service in the Women’s College Hospital Reproductive Life Stages Program or other similar programs?

**3. Overall Comments**:

1. Any final comments on eMBC?
2. Did anything surprise you?
3. How did the interview feel for you?

***Probes (can be used throughout if needed):***

- *Can you give me a specific example?*
- *Can you explain your answer?*
- *In what way?*
- *How did you understand that?*
- *What does that mean to you?*

***Redirection (can be used throughout if needed):***

- *I understand there are issues with Module X but in the interest of time I would like to move on to some other questions to make sure we get to talk about the other modules as well.*

## Wrap up

Finish the interview by thanking the participant for their time.

# eMBC Qualitative Interview Guide for Patients

**Implementation of technology-enabled (Electronic) Measurement Based Care (eMBC) for Perinatal Depression and Anxiety: Qualitative Study**

Interview Guide – Patient Participants

The interview guide was adapted from a template created by (Cuperfain et al., 2021).

**Venue**: Interviews will be conducted at Women’s College Hospital, by telephone, video or in-person, whichever works best and provides sufficient privacy. The exact time and location will depend on room availability and will be communicated to participants in advance.

**Duration**: Interviews will last approximately 30-60 minutes or as long as it takes for the participant to complete their thoughts. If the participant is tried, let them take a break.

**Number of Facilitators**: Each interview will be conducted by a member of the research team.

## Set Up

1. Introduce yourself and the purpose of the interview, e.g., “I am a physician/investigator/research assistant working on a research study about electronic Measurement Based Care (eMBC), a new model of care that involves the regular completion of symptom scales to measure depression and anxiety. The main purpose of this interview is to understand your perspective and experience with electronic measurement-based care or eMBC in pregnancy and postpartum.
2. Review the key content in the consent form (e.g., confidentiality and anonymity, the participant’s right to withdraw and to delete data) and offer to answer any questions the participant may have.
3. Explain the need for audio-recording and obtain approval from all participants. [Remember to bring your recorder and to check it for proper functioning, including sufficient battery life, memory space for recording].

When the audio-recorder is turned on, state “For the purpose of the recording, I would like to state that today is *(date)* and this is my *(time)* interview with *(participant ID).*”

## Interview Discussion

**1. Background:**

1. Can you tell me a little bit about yourself? Your background?
2. How did you come to be receiving care with Women College Hospital? Have you been diagnosed with anything?

**2. Open Exploration**

1. Start with a brief review of eMBC

The main purpose of this interview is to understand your perspective, experience and feelings about electronic measurement-based care or eMBC. Prior to beginning this interview, I would like to provide a brief overview of what eMBC is.

Electronic Measurement Based Care (eMBC) is a model of care in that involves the regular completion of questionnaires (also known as symptom scales). Within our study, you would have completed a few questionnaires through MyHealthRecord ideally before your appointment with your psychiatrist at Women’s College Hospital. Your answers to these questionnaires are automatically linked with your electronic medical record to potentially support patient provider collaborative review.

We are most interested in your personal experience and perspective on eMBC. You can start with whatever you want to talk about first.

1. The main purpose of the open exploration part is to allow participants to freely express themselves. The participant should decide what they want to talk about based on what is important to them.
2. Use more probes to invite participants to elaborate on certain topics.
3. Try and jot down notes during the interview to help keep track of what has been said
4. Once you believe the open exploration is complete, try to summarize key points of the conversation and thank them for sharing.
5. Introduce the participants to the structured exploration part of the interview “Next, I have a few specific questions I am going to ask about your experience and perspective on eMBC”

**3. Structured inquiry:** Ask participants about specific features of eMBC. For each feature, remember to encourage the “think aloud” method and ask:

- *How do you feel when you see this?*
- *What was your initial reaction to this?*
- *What do you think this feature/content is trying to accomplish?*

1. What kinds of treatment are you aware of for pregnant/postpartum people with depression and anxiety?
2. What kinds of treatment have you received? What are your experiences with treatment?
3. In general, what are your experiences and comfort level with technology?
   1. What kinds of virtual or web-based treatments do you have experience with (e.g. online therapy, mobile mental health apps)?
4. What were your initial reactions to eMBC? What were your thoughts about how eMBC may impact your mental health and/or treatment?
   1. What drew you to the eMBC study?
5. What do you think are the goals of eMBC and what is your view/opinion of them?
   1. Did you have the goals of eMBC explained to you? By who?
6. How was your experience learning how to use eMBC (i.e. completing symptom scales on MyHealthRecord)?
   1. What are your thoughts about the training you received?
   2. Do you have any suggestions for how we can improve training?
7. Overall, how was your experience using eMBC as a part of your treatment in the RLS program?
   1. What did you like most about eMBC? What were the greatest benefits?
   2. What did you like least about eMBC? What were the greatest challenges?
      - How did you navigate these challenges?
      - What would have helped address any challenges you may have faced in utilizing eMBC?
      - Any Technical issues?
      - What are your views on possible risks of eMBC?
8. How did completing the symptom scales impact you or your daily activities (i.e. being pregnant or postpartum with infant)?
   1. Do you feel that eMBC impacted your treatment or recovery? If you have previously received treatment for depression or anxiety without eMBC, how does eMBC compare?
   2. Do you think eMBC will improve the quality of life of users?
9. Were your scales discussed in your clinical appointments with your psychiatrist? If so, how was that? If not, do you think anything got in the way?
10. Do you have any suggestions about how we can improve eMBC?
    1. Here are the questionnaires from the study.
       - How did you feel about completing these questionnaires?
       - Did these scales address certain parts of your diagnosis (e.g., depression or anxiety)? Did they miss any?
       - Did you find that some scales were more relevant to you than others?
    2. Is there anything specific you would add or change? (e.g. content or delivery)
11. In a perfect world what you like to see happen with eMBC integration?
12. Would you recommend eMBC to other women who are pregnant or postpartum with similar mental health problems to you own? Do you think they would benefit from using eMBC?
13. Do you have any other suggestions about how eMBC could best be integrated with your care?

**4. Overall Comments**:

1. Any final comments on eMBC?
2. Did anything surprise you?
3. How did the interview feel for you?

***Probes (can be used throughout if needed):***

- *Can you give me a specific example?*
- *Can you explain your answer?*
- *In what way?*
- *How did you understand that?*
- *What does that mean to you?*

***Redirection (can be used throughout if needed):***

- *I understand there are issues with Module X but in the interest of time I would like to move on to some other questions to make sure we get to talk about the other modules as well.*

## Wrap Up

Finish the focus group by thanking the participants for their time.
