# Supplementary material for: Electronic Measurement-based care (eMBC) for perinatal depression and anxiety: a pilot randomized controlled trial
Source: BMC Psychiatry. 2025 Apr 29;25:437. doi: 10.1186/s12888-025-06876-3 (PMC12042506; doi:10.1186/s12888-025-06876-3)
Supplement: Supplementary file 3 — Supplementary Material 3 [file 12888_2025_6876_MOESM3_ESM.docx]

Appendix A: Provider and Patient Training Guides

eMBC Training Guide for Providers v6; 20-Jan-2023; Patients v4; 20-Jan-2023

Contents

[eMBC Training Guide for Providers 2](#_Toc185419590)

[What is Measurement Based Care? 2](#_Toc185419591)

[What Questionnaires will be used? 2](#_Toc185419592)

[Interpreting Scores-EPDS 3](#_Toc185419593)

[Interpreting Scores- PROMIS QOL 3](#_Toc185419594)

[Interpreting Scores- ASCQ-ME Social Functioning 4](#_Toc185419595)

[Interpreting Scores- FISBER 4](#_Toc185419596)

[How will it work? 5](#_Toc185419597)

[Safety Planning 5](#_Toc185419598)

[Setting eMBC Flag 6](#_Toc185419599)

[View eMBC Questionnaire Results 8](#_Toc185419600)

[eMBC Training Guide for Patients 13](#_Toc185419601)

[What is Measurement-Based Care (MBC)? 13](#_Toc185419602)

[How Does eMBC Work? 13](#_Toc185419603)

[Safety Planning 14](#_Toc185419604)

[How to View and Complete Questionnaires 15](#_Toc185419605)

[Review with Health Care Provider 17](#_Toc185419606)

# eMBC Training Guide for Providers

Thank you for agreeing to participate in the eMBC study!

The following training guide will provide you with instructions on how to receive your patient’s eMBC questionnaire responses prior to their upcoming visit. If you have any questions, please contact the research team.

## What is Measurement Based Care?

**Measurement Based Care (MBC)** is a model of care in which systematic tracking of psychiatric symptoms, functioning and adverse treatment effects, coupled with regular patient-provider collaborative review, enables more effective and efficient symptom reduction.

The Department of Psychiatry at Women’s College Hospital is doing a research study to test the feasibility of MBC in pregnant and postpartum women with depression and anxiety. It is hoped that by helping to tailor treatment to a woman’s individual needs (e.g. helping identify when medication is needed and what dose is effective), MBC may increase remission rates in this population.

To address previously-identified implementation barriers to MBC, we will use **electronic MBC (eMBC)** where patients track their symptoms digitally prior to their visits, with results integrated into the electronic health record (EPIC) for real-time evaluation in session with their provider.

## What Questionnaires will be used?

eMBC questionnaires in the study will be:

1. Symptoms:

- Edinburgh Postnatal Depression Scale

1. Functioning:

- PROMIS Neuro QOL – Ability to Participate in Social Roles and Activities – Short Form
- PROMIS ASCQ-Me Social Functioning – Short Form

1. Adverse effects of treatment:

- Adapted FIBSER Scale (patient only completes if taking an antidepressant)

## Interpreting Scores-EPDS

- The EPDS is a 10-item symptom scale designed for the perinatal population and measures depressive and anxious symptoms experienced in the past 7-days
- Scores range from 0-30
- Score interpretation:
  - - **Less than 8**: Depression not likely
    - **9-11**: Depression possible
    - **12-13**: Fairly high possibility of depression
    - **14 and above**: Probable depression
    - Positive score (above 0) on item 10: Further assessment and intervention as appropriate

## Interpreting Scores- PROMIS QOL

- The PROMIS QOL is a measure of the patient’s ability to participate in social roles and activities
- Items are scored on a 5-point scale from (1) Never to (5) Always, with higher scores indicating higher social functioning
- T-score distributions rescale raw scores into standardized scores with a mean of 50 and a standard deviation (SD) of 10. Thus, a person who has a T-score of 60 is one SD above the average
- The chart to the right has converted each raw score into a T-Score for interpretation. 95% percent of people are within the T-scores shown below


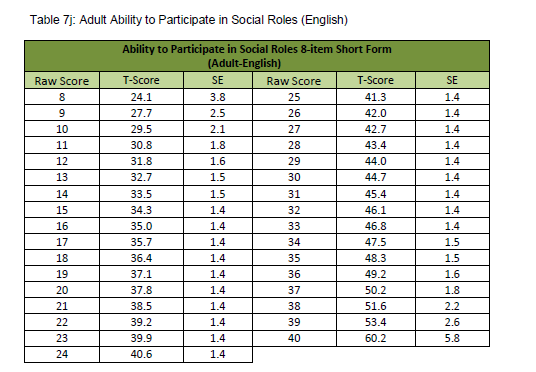


## Interpreting Scores- ASCQ-ME Social Functioning

- The ASCQ-ME is a scale of a disease’s impact on a patient’s ability to function socially
- Items rating social functionality are scored on a 5-point scale from (5) Never to (1) Always, with higher scores indicating higher social functioning
- T-score distributions rescale raw scores into standardized scores with a mean of 50 and a standard deviation (SD) of 10. Thus, a person who has a T-score of 60 is one SD above the average
-
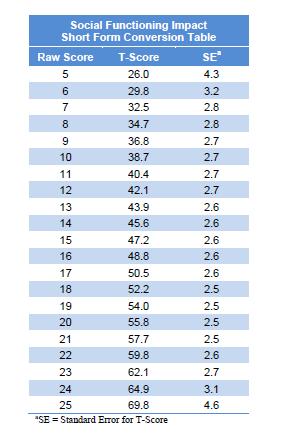
The chart to the right has converted each raw score into a T-Score for interpretation. 95% percent of people are within the T-scores shown below

## Interpreting Scores- FISBER

- The Frequency, Intensity, Burden of Side Effects Rating (FIBSER) is a 3-item scale to assess side effects from antidepressant treatment.
- For each item, and **especially item #3** (burden):
  - **0-2 = none to mild**
    - Likely recommend no change in medication due to side effects
  - **3-4 = moderate to marked**
    - May need to consider addressing side effects, especially if persistent, e.g. by changing dose or adding antidote
  - **5-6 = severe interference with activities or unable to function**
    - Highly likely that dose should be decreased or medication switched

## How will it work?

1. If you have a patient who is 18 years of age of older, pregnant or postpartum, and in whom you are considering antidepressant use, please refer them to the study by emailing: [emailaddressTBD@wchospital.ca](mailto:emailaddressTBD@wchospital.ca)
2. The patient will be evaluated for their eligibility to participate in the study, and then consenting participants will be randomized to usual care, or to the eMBC intervention. We will notify you if your patient is randomized to eMBC.
3. For patients randomized to the eMBC intervention:
   - - - The **research staff** will show you how to configure MyHealthRecord so that patients receive the eMBC questionnaires prior to their upcoming appointment.
       - Participants will fill out eMBC questionnaires on MyHealthRecord.
       - The questionnaires will automatically be “pushed” into EPIC so that you will see them in the “Synopsis” section.
       - At your appointment with the patient, you will then review the results of the questionnaires (e.g. symptom improvement, response to treatment). and subsequently re-evaluate the treatment plan as appropriate.
       - Please document in your note whether or not you reviewed the scales with the patient.

## Safety Planning

- Participants will be informed that their responses to the eMBC questionnaires will **NOT** **be monitored in real-time by the psychiatrist.**
- Participants will sign a consent form stating that the psychiatrist will **NOT** be able to respond to a mental health crisis (e.g. suicidality) or other concerns that come up on the eMBC questionnaire in real-time. Rather, participants and providers will review the results of the questionnaires with their psychiatrist during their appointment.
- If the patient has a very high score on their questionnaire, or is expressing thoughts about harming themselves, they will receive a message within MyHealthRecord with recommendations for how to receive emergency care if they need it prior to their appointment (see next page).

If a patient has a very high score on their questionnaire, or are expressing thoughts about harming themselves, they will receive the following response after completing their questionnaires:


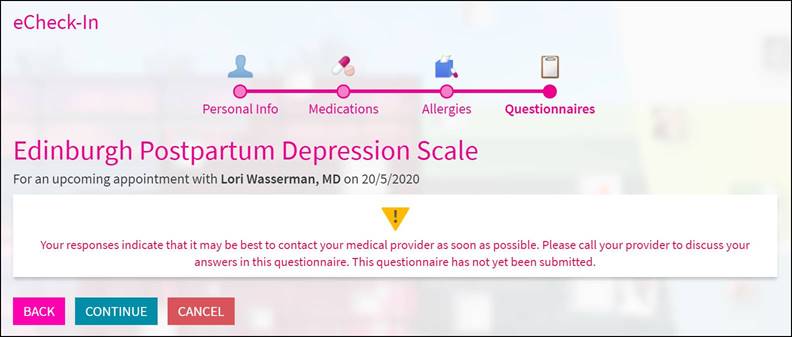


**They will be instructed to follow the recommended instructions listed on MyHealthRecord**:

**“**Your responses indicate that it might be best to contact your health care provider as soon as possible to discuss the results of your questionnaire. The Toronto Distress Centre can be reached 24 hours a day at 416-408-4357 or 408-HELP. If this is an emergency, please go to your nearest emergency department or call 911. **Please press continue to submit your results.”**

## Setting eMBC Flag

The following pages demonstrate how to set the eMBC flag in EPIC

Navigate to the **FYI activity tab** of the chart for the patient enrolled in the eMBC study.


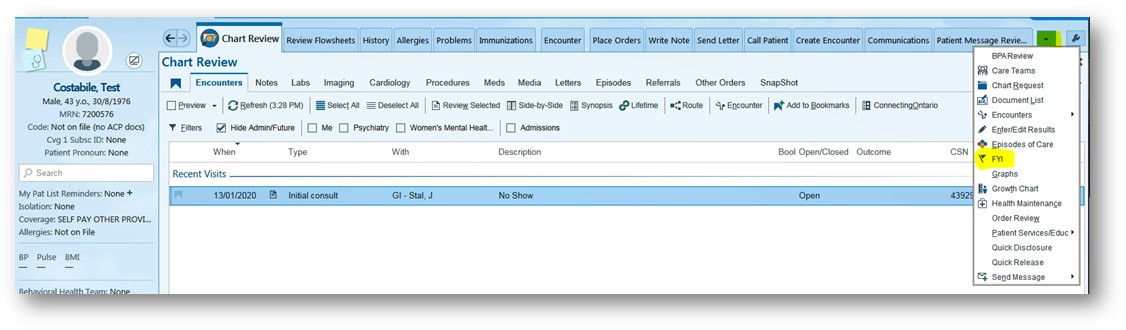


Click **New Flag**.


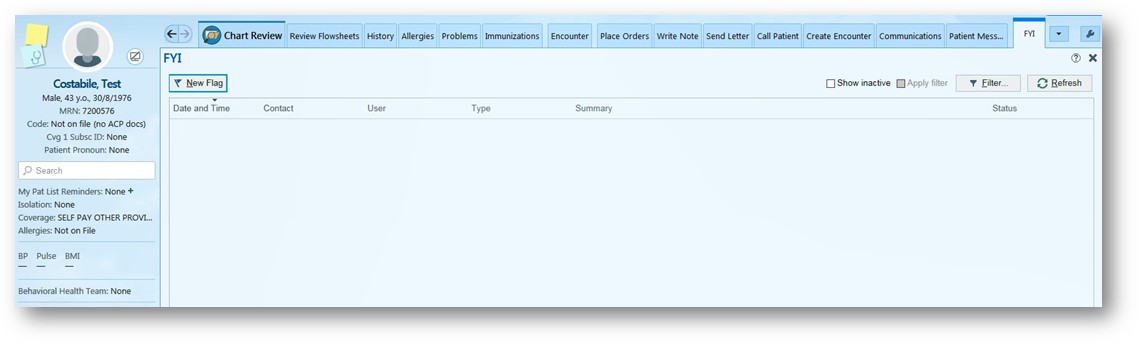


Type **“eMBC Study”** into the flag type search field. Then click **Accept**.


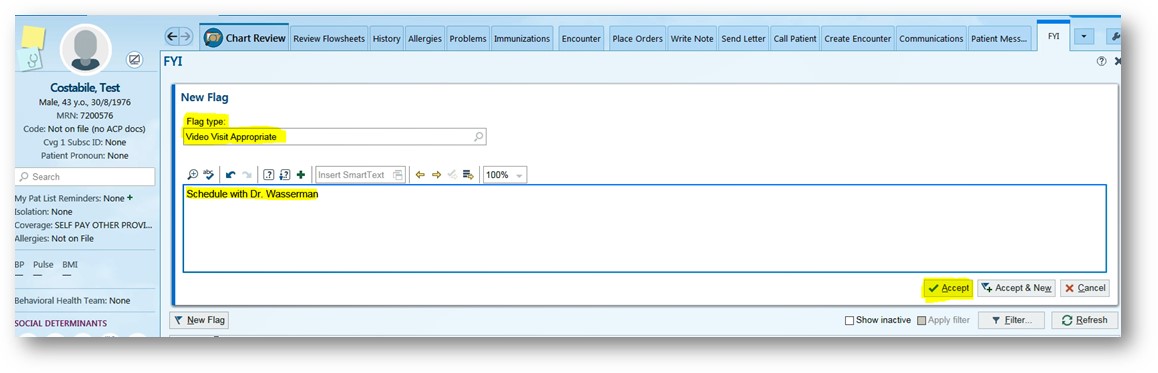


**eeMBC Study**

To deactivate the flag after the study, navigate to the FYI tab in their chart, select the flag in the list and click **Deactivate**.


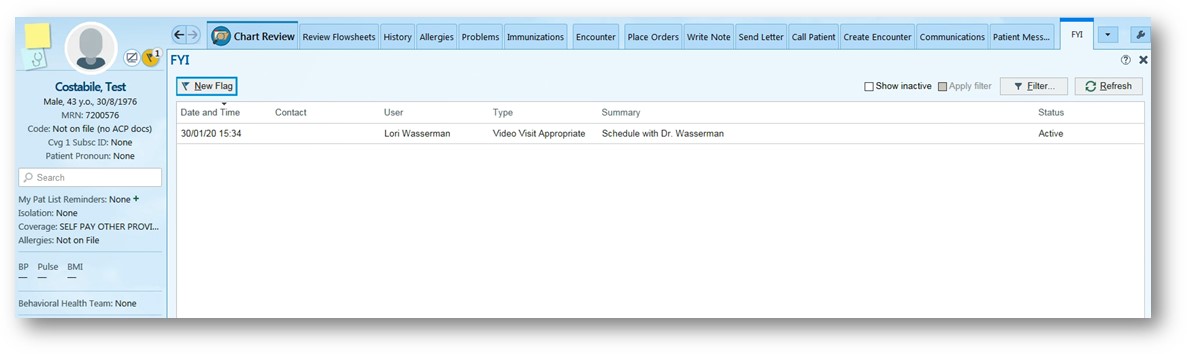


The flag will be listed in the FYI tab and indicated in the sidebar with the orange flag icon. If you don’t see it, you can refresh the chart.

## View eMBC Questionnaire Results

The following pages demonstrate how the results of the eMBC questionnaires will come into your mailbox prior to the clinical encounter.


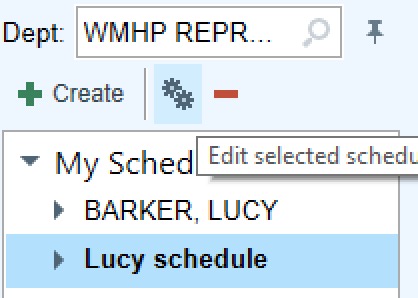


On your main page, select the cog wheels above your schedule to go to display settings for schedule.

In the **Available Columns**, click on **Assigned QNR** then click **Add Column**.


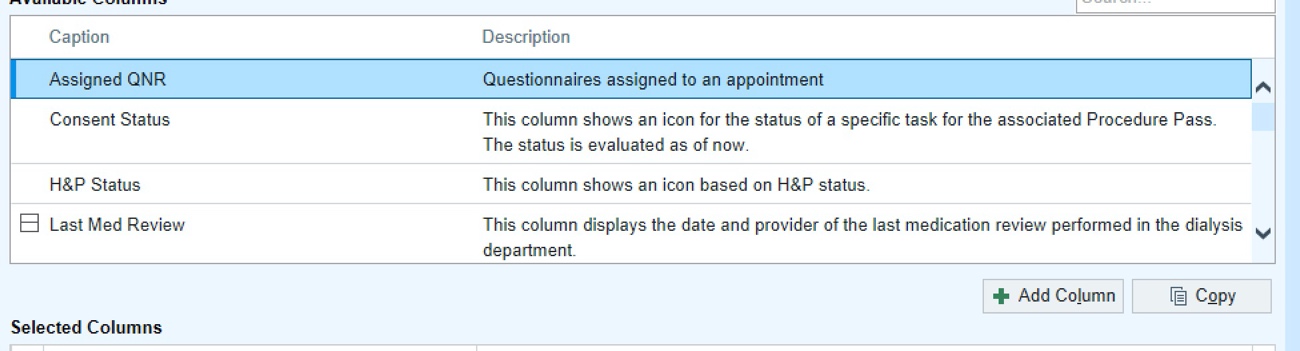


Repeat for **QNR Status**.


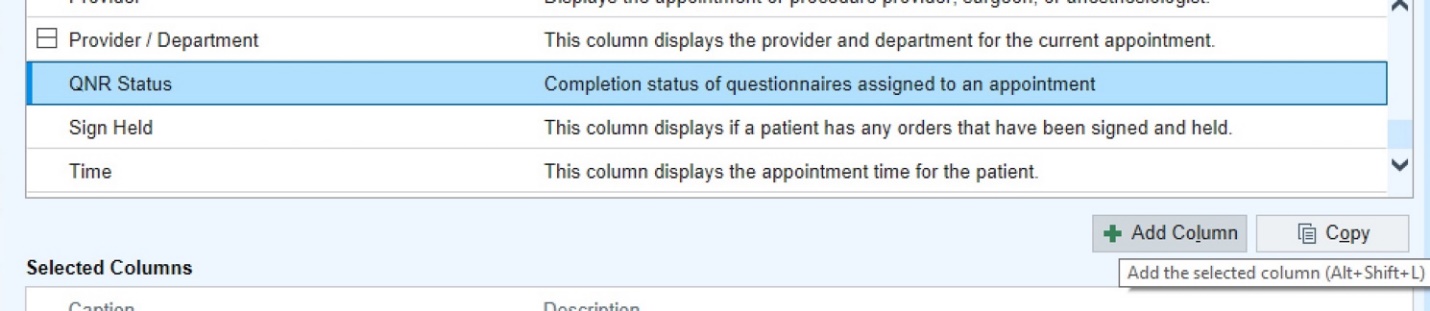


Under **Selected Columns**, use the up and down arrows in the bottom right corner to move **Assigned QNR and QNR Status** to where you want so they are visible. Then click **Accept**.


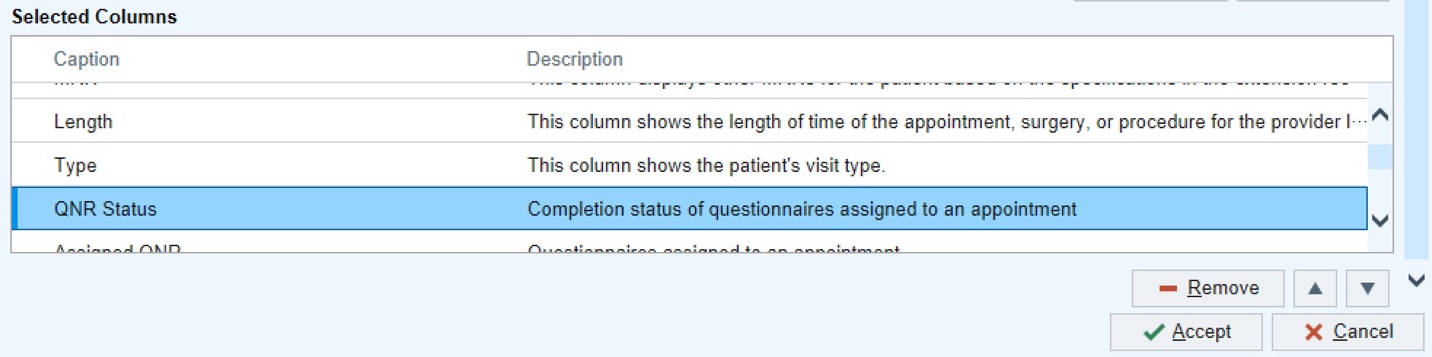


You should then see the columns above your schedule.


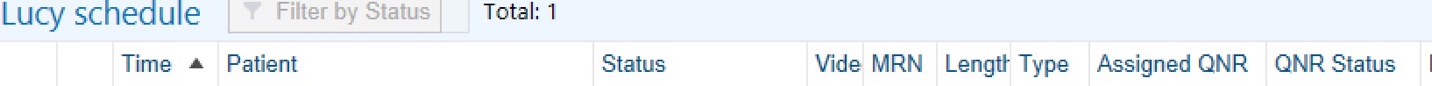


**Viewing the questionnaires with Synopsis**

To view the patient’s scores in the clinical encounter, go to the “**Synopsis**” tab.


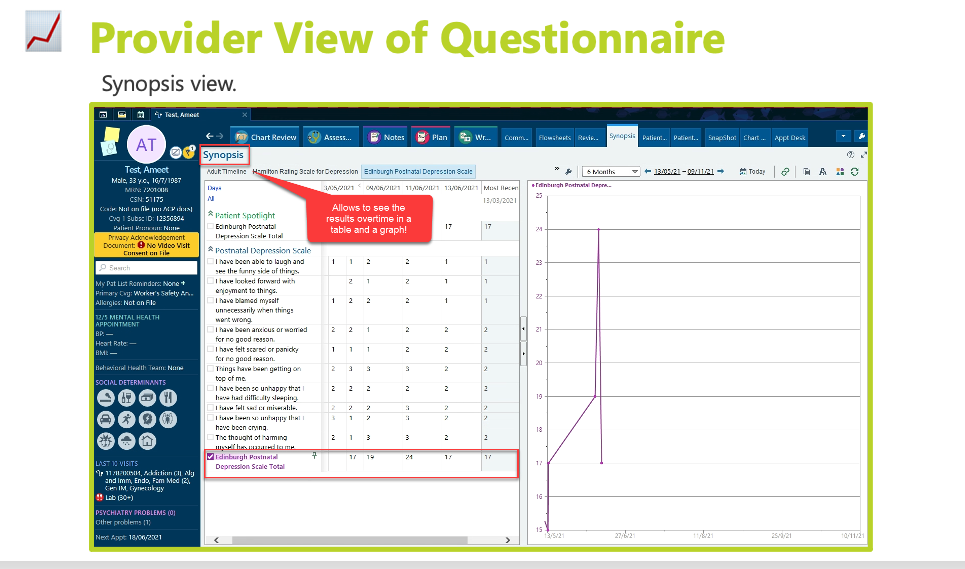


Click on the **[>>]** button.


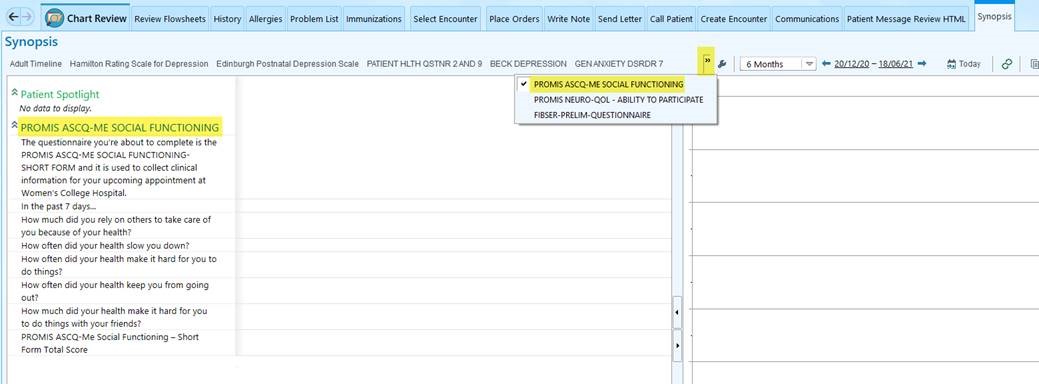


From the **drop-down** options select the **questionnaire** you’d like to view.


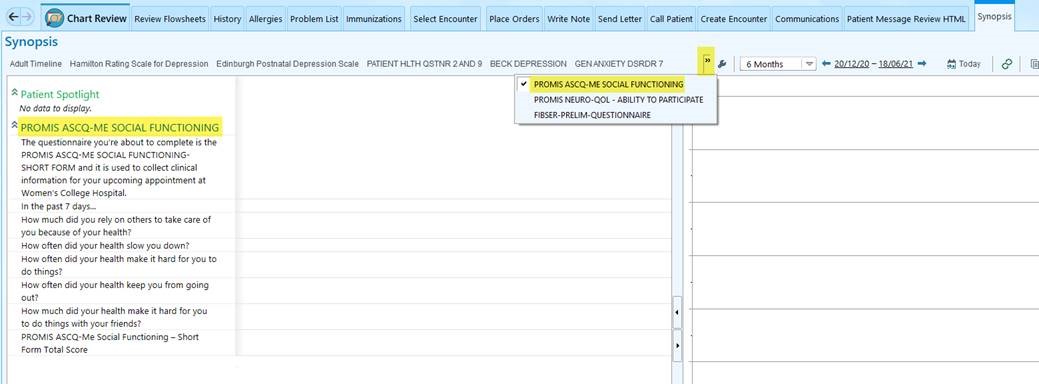


**Graphing Flowsheet results over time**

The questionnaire score values appear in a table on the **left** of the page
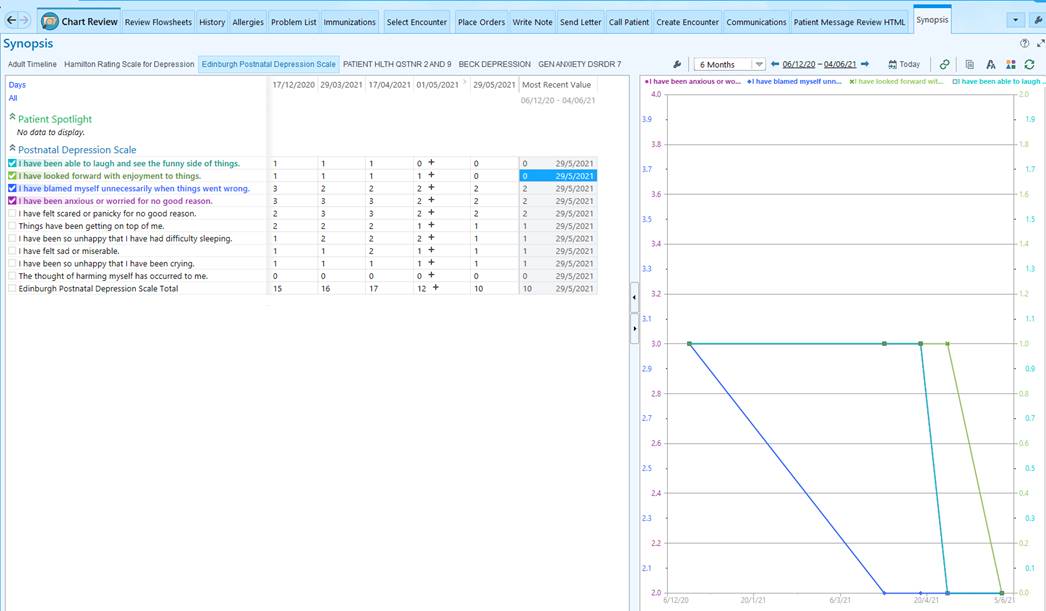


The graph view of the score values is always present to the **right** of the table. You can select **up to 4** questions or score values to graph.


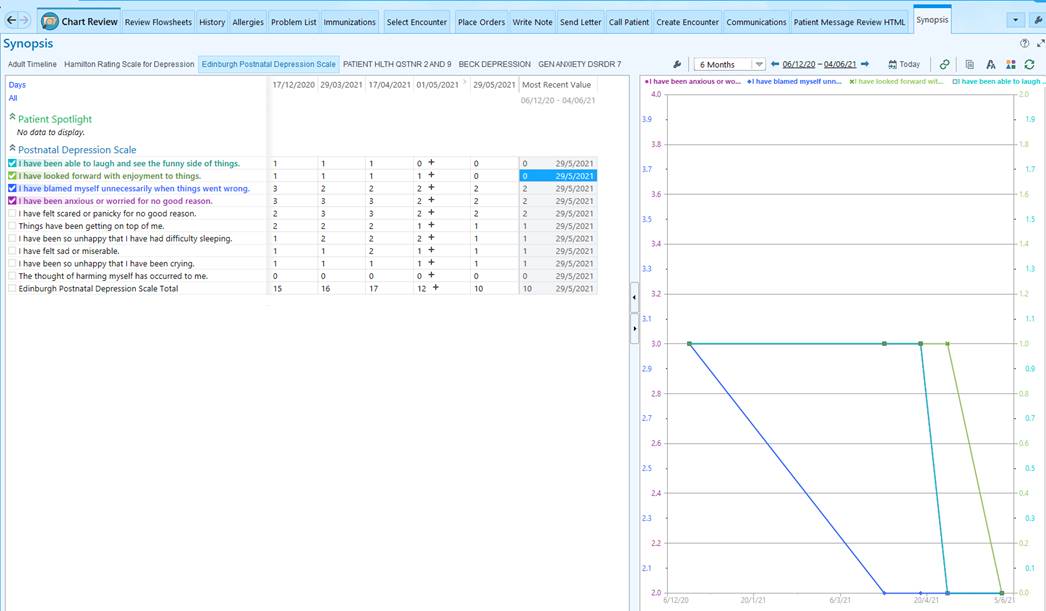


**Editing the order of Flowsheets that appear in the Synopsis activity**

To change the order of the flowsheet-report list in the Synopsis activity, click the **wrench icon**.


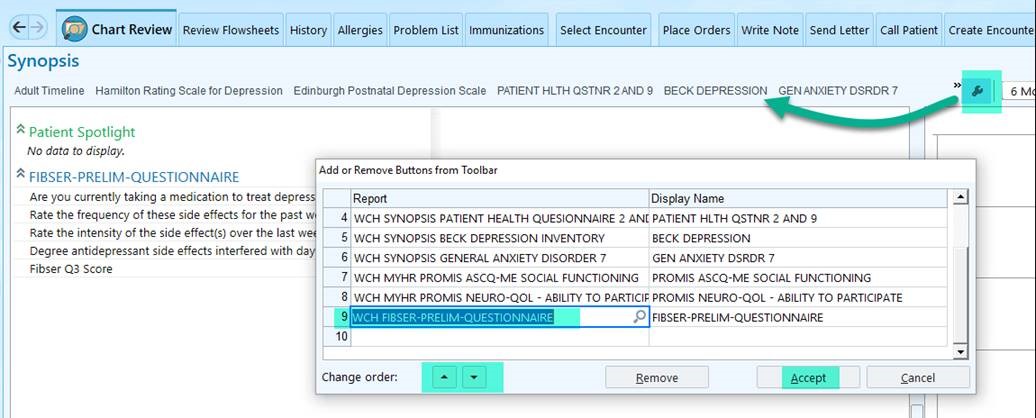


Select the questionnaire and use the **up and down buttons** to move the questionnaire up or down in the list.


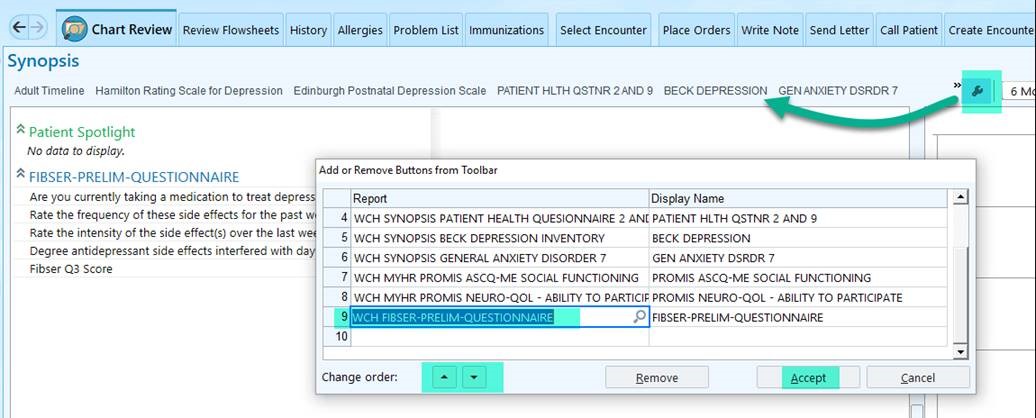


# eMBC Training Guide for Patients

Thank you for agreeing to participate in the eMBC study!

The following training guide will provide you with instructions on how to view and complete your eMBC questionnaires prior to your upcoming visit. If you have any questions, please contact the research team at:

## What is Measurement-Based Care (MBC)?

**Measurement Based Care (MBC)** is a new model of care that involves the regular completion of questionnaires prior to your next scheduled appointment, and patient-psychiatrist collaborative review of the results of the questionnaires during the appointment.

MBC allows for ongoing assessment that can contribute to more appropriate provision of treatment and more rapid improvements in your mental health.

MBC can help you:

1. Be better informed about your symptoms
2. Be more supported and involved in your treatment
3. Be able to more easily make decisions about treatment that feels right for you.

To ease the process of regularly completing questionnaires, we will use **electronic MBC (eMBC),** where you can complete your questionnaires online prior to your next scheduled appointment, with your results integrated into your electronic health record for real-time evaluation during your appointment.

## How Does eMBC Work?

- You will receive a notification prior to your upcoming appointment that the eMBC questionnaire is waiting for you to complete
- You will complete the eMBC questionnaire **prior** to your upcoming appointment
- During your appointment, you and your psychiatrist will collaboratively review the results of your eMBC questionnaires

**Important Disclaimer:**

Responses to the eMBC questionnaires that you fill out during the study will **NOT** be monitored in real-time by your health care provider.

So, we will not be able to immediately respond to a mental health crisis (for example, suicidal thoughts) or other concerns that come up on the eMBC questionnaires.

*You will review the results of your eMBC questionnaire with your health provider on the day of your appointment.*

If you have a very high score on your eMBC questionnaire, or you are expressing thoughts about harming yourself, you will receive a message within MyHealthRecord with recommendations for how to receive emergency care if you need it prior to your appointment (see next page).

## Safety Planning

If you have a very high score on your eMBC questionnaire, or you are expressing thoughts about harming yourself, you may receive the following response after completing your eMBC questionnaires:


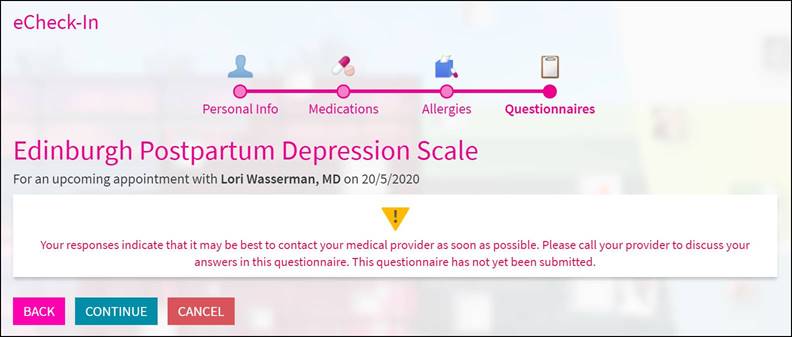


**Please follow recommended instructions listed on MyHealthRecord:**

Your responses indicate that it might be best to contact your health care provider as soon as possible to discuss the results of your questionnaire. The Toronto Distress Centre can be reached 24 hours a day at 416-408-4357 or 408-HELP. If this is an emergency, please go to your nearest emergency department or call 911. **Please press continue to submit your results.**

## How to View and Complete Questionnaires

48 hours before your appointment, you will get a notification in your email inbox to check-in for your appointment. The notification check-in is also your reminder to complete your eMBC questionnaires.

There are **two** places in MyHealthRecord where you can access your eMBC questionnaires before your appointment:

**OPTION 1:** You can navigate to the eMBC questionnaires through the MyHealthRecord Homepage

OR

**OPTION 2:** You can navigate to the eMBC questionnaires through MyHealthRecord Menu > My Record > Questionnaires tab

**Option 1. MyHealthRecord Homepage**

**View Instructions Notification in MyHealthRecord Homepage**

- Clicking on “Begin Visit” or “eCheck-In” will take you to a page where you will see the eMBC questionnaires


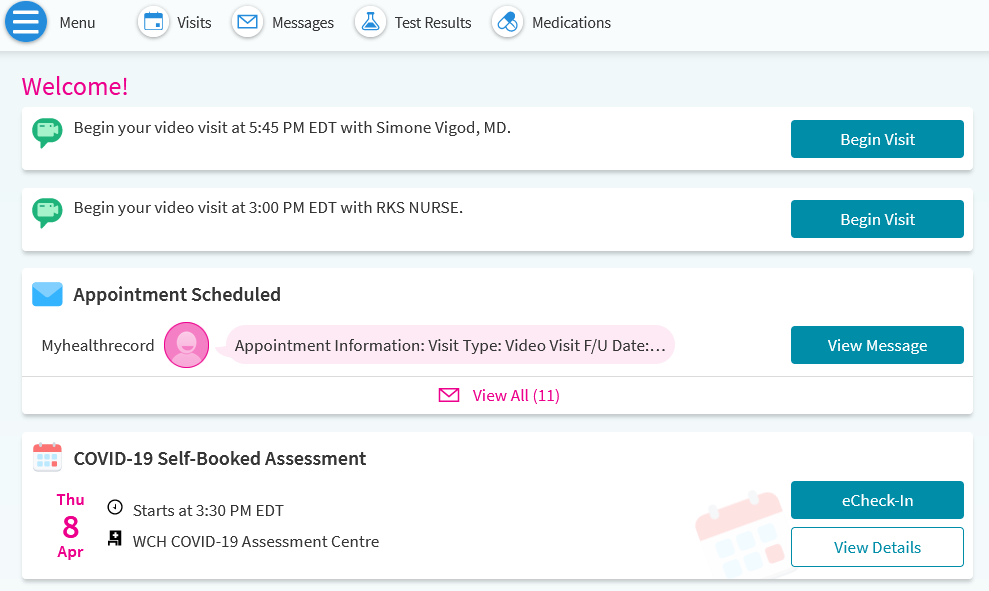


**Option 2. Menu > My Record > Questionnaires**


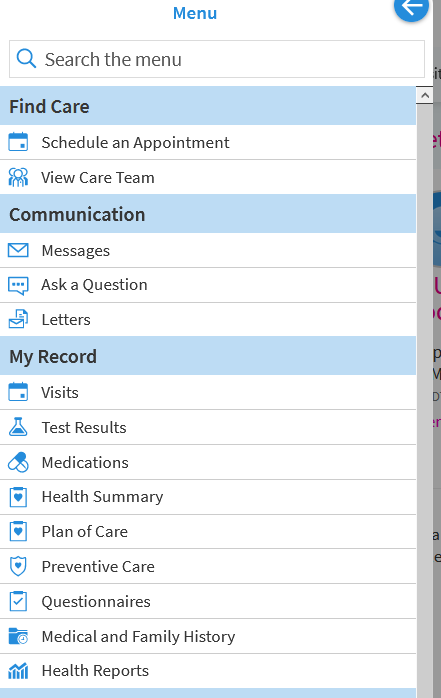


**Complete the Questionnaires (5 minutes)**

You may have a number of eMBC questionnaires to complete before your appointment (max 5 minutes total).

In each eMBC questionnaire, you will be asked to select the most appropriate answer.

You can continue to the next set of questions or save and finish later.


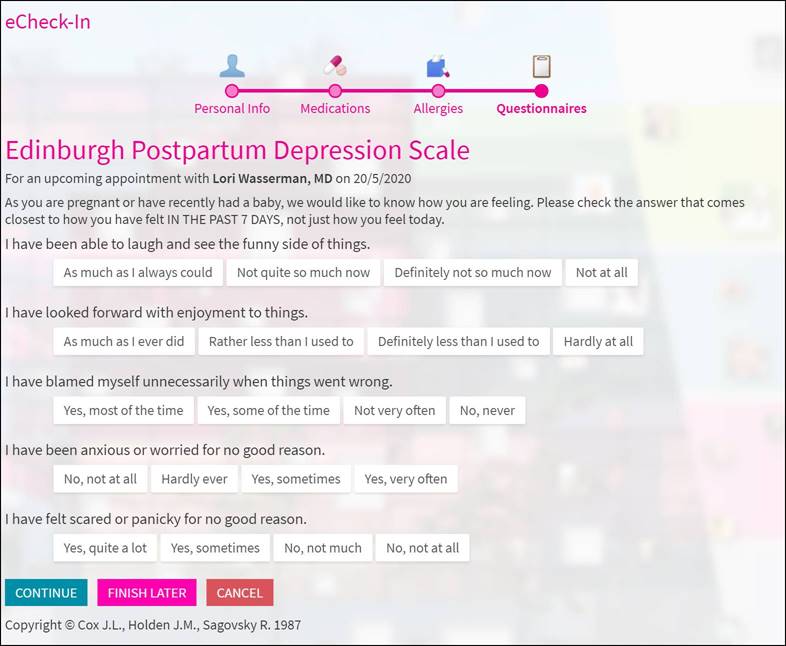


**How to Review and Submit**

You will be prompted to review your answers to the eMBC questionnaire before submitting them.

You may modify your answers before submission.

Click “Submit” when you are ready to submit your answers.


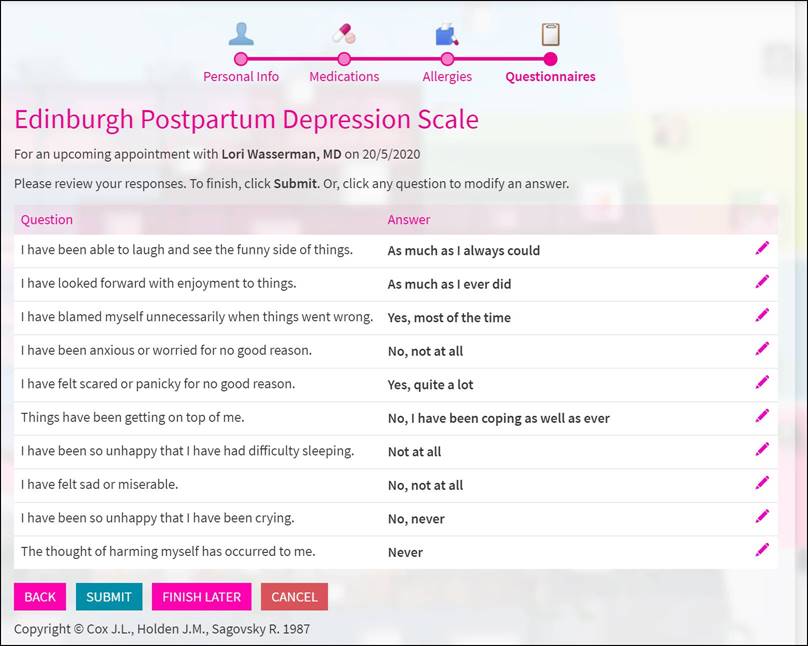


## Review with Health Care Provider

During your appointment, **you and your psychiatrist will collaboratively** review your eMBC questionnaires.

This may involve reviewing any of the following:

- The results of your eMBC questionnaires
- Any changes that can be made to your treatment plan to improve your mental health
