# Supplementary material for: Electronic Measurement-based care (eMBC) for perinatal depression and anxiety: a pilot randomized controlled trial
Source: BMC Psychiatry. 2025 Apr 29;25:437. doi: 10.1186/s12888-025-06876-3 (PMC12042506; doi:10.1186/s12888-025-06876-3)
Supplement: Supplementary file 4 — Supplementary Material 4 [file 12888_2025_6876_MOESM4_ESM.docx]

**Table S1.** Baseline characteristics for eMBC providers, presented as N (%) unless otherwise specified

| Variable | Provider (N=9) |
| --- | --- |
| Age Category |  |
| <30 | 0 |
| 31-35 | 3 (33.3) |
| 36-40 | 3 (33.3) |
| 41-45 | 3 (33.3) |
| 46-50 | 0 |
| 50-60 | 0 |
| 60+ | 0 |
| Number of Years Practicing* |  |
| <1 | 1 (12.5) |
| 1-2 years | 0 |
| 3-5 years | 4 (50.0) |
| 6-10 years | 2 (25.0) |
| 11-15 years | 1 (12.5) |
| 16-20 years | 0 |
| 20+ years | 0 |
| Familiarity with Technology in General* |  |
| Excellent | 1 (12.5) |
| Very good | 2 (25.0) |
| Good | 4 (50.0) |
| Fair | 1 (12.5) |
| Poor | 0 |
| Familiarity with Technology in your Clinical Practice |  |
| Excellent | 1 (11.1) |
| Very good | 1 (11.1) |
| Good | 6 (66.7) |
| Fair | 1 (11.1) |
| Poor | 0 |
| How Often do you Use Technology in your Clinical Practice |  |
| Never | 0 |
| Rarely | 0 |
| Sometimes | 0 |
| Often | 2 (22.0) |
| Very Often | 7 (77.8) |
| How Often do you Use the Following in your Clinical Practice |  |
| Email |  |
| Never | 1 (8.3) |
| Rarely | 3 (33.3) |
| Sometimes | 0 |
| Often | 0 |
| Very Often | 6 (66.7) |
| Telephone |  |
| Never | 0 |
| Rarely | 0 |
| Sometimes | 1 (11.1) |
| Often | 3 (33.3) |
| Very Often | 5 (55.6) |
| Mobile devices (e.g. mobile health application) |  |
| Never | 2 (22.2) |
| Rarely | 2 (22.2) |
| Sometimes | 3 (33.3) |
| Often | 0 |
| Very Often | 2 (22.2) |
| Electronic Health Records (EHRs) |  |
| Never | 0 |
| Rarely | 0 |
| Sometimes | 0 |
| Often | 0 |
| Very Often | 9 (100) |
| Live Interactive Video-Conferencing |  |
| Never | 0 |
| Rarely | 0 |
| Sometimes | 0 |
| Often | 0 |
| Very Often | 9 (100) |
| Social Media (i.e. Facebook, Twitter) |  |
| Never | 9 (100) |
| Rarely | 0 |
| Sometimes | 0 |
| Often | 0 |
| Very Often | 0 |
| Comfortability Using Technology in your Clinical Practice |  |
| Not very comfortable | 0 |
| Slightly comfortable | 0 |
| Somewhat comfortable | 1 (11.1) |
| Moderately comfortable | 7 (77.8) |
| Completely comfortable | 1 (11.1) |
| Confident you are in Using Technology in your Clinical Practice |  |
| Not confident at all | 0 |
| Slightly confident | 0 |
| Somewhat confident | 3 (33.3) |
| Moderately confident | 5 (55.6) |
| Completely confident | 1 (11.1) |
| Satisfaction with the Use of Technology in your Clinical Practice |  |
| Extremely dissatisfied | 0 |
| Dissatisfied | 0 |
| Neither dissatisfied or satisfied | 2 (22.2) |
| Satisfied | 6 (66.7) |
| Extremely Satisfied | 1 (11.1) |
| Have you used Measurement Based Care in your Clinical Practice Prior to this Research Study? |  |
| Yes | 6 (66.7) |
| No | 3 (33.3) |

*One observation missing
